# Armadillo Motifs Involved in Vesicular Transport

**DOI:** 10.1371/journal.pone.0008991

**Published:** 2010-02-01

**Authors:** Harald Striegl, Miguel A. Andrade-Navarro, Udo Heinemann

**Affiliations:** 1 Max-Delbrück-Centrum für Molekulare Medizin, Berlin, Germany; 2 Institut für Chemie und Biochemie, Freie Universität Berlin, Berlin, Germany; Griffith University, Australia

## Abstract

Armadillo (ARM) repeat proteins function in various cellular processes including vesicular transport and membrane tethering. They contain an imperfect repeating sequence motif that forms a conserved three-dimensional structure. Recently, structural and functional insight into tethering mediated by the ARM-repeat protein p115 has been provided. Here we describe the p115 ARM-motifs for reasons of clarity and nomenclature and show that both sequence and structure are highly conserved among ARM-repeat proteins. We argue that there is no need to invoke repeat types other than ARM repeats for a proper description of the structure of the p115 globular head region. Additionally, we propose to define a new subfamily of ARM-like proteins and show lack of evidence that the ARM motifs found in p115 are present in other long coiled-coil tethering factors of the golgin family.

## Introduction

The armadillo (ARM) repeat motif is present in a variety of proteins. It was first described in the *Drosophila* segment-polarity gene product armadillo [1], the mammalian homolog of β-catenin that is essential for cadherin-based cell adhesion and Wnt/Wingless growth factor signaling. Furthermore, it functions to bridge the cytoplasmic domain of cadherins to α-catenin and the actin cytoskeleton [2], [3] and is associated to multiple diseases including cancer [4]–[7].

**Figure 1:**
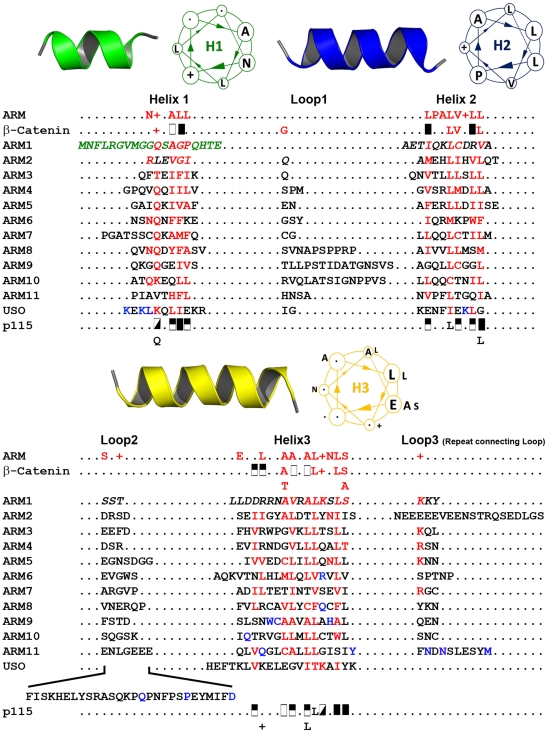
ARM-motif consensus and structure-based sequence alignment of human p115^GHR^. On the top of the alignment the cartoon and helical wheel representation of isolated ARM-repeat helices are shown. Each repeat is composed of three helices that are displayed in green (H1), blue (H2), and yellow (H3). The universal ARM-repeat consensus sequence derived from the alignment of five ARM-repeat proteins [8] is shown beneath as well as the consensus sequence for β-catenin [11] followed by the single ARM-repeat sequences of p115^GHR^ and, at the bottom of the alignment, the consensus sequence for p115^GHR^. Residues comprising H1, H2 and H3 in each repeat are separated by their connecting loop regions. Italicized residues are not present in the X-ray structure of human p115^GHR^ [13] and derived from the structure of the bovine homolog [14]. Residues shown in green are missing from both the human and bovine p115^GHR^ structure. Conserved residues that define the ARM-consensus motif are highlighted in red. Structural positions with strong preferences for a given amino acid or group of amino acids are shaded with the following symbols: half-closed box = general hydrophobic; open box = small hydrophobic; diagonal-filled box = hydrophilic; closed box = large hydrophobic; (+) = basic. In the consensus sequence, the single-letter code is listed at the bottom if the residue is present in at least six of twelve repeats. Residues that mediate contacts (hydrogen bond or salt bridge) between the USO repeat and the USO-domain helix H3 are highlighted in blue.

The presence and arrangement of ARM motifs differ in various proteins, and it was suggested that these linked units comprise a structural domain described by a universal consensus sequence (Figure 1) [8]. The number of tandem ARM repeats in an ARM fold ranges from 6 to 12. Based on the organization of their ARM motifs, three major subfamilies of ARM-like proteins are distinguished, namely the classical catenins, the p120^ctn^ related catenins and the proteins involved in nuclear import [9], [10].

**Figure 2:**
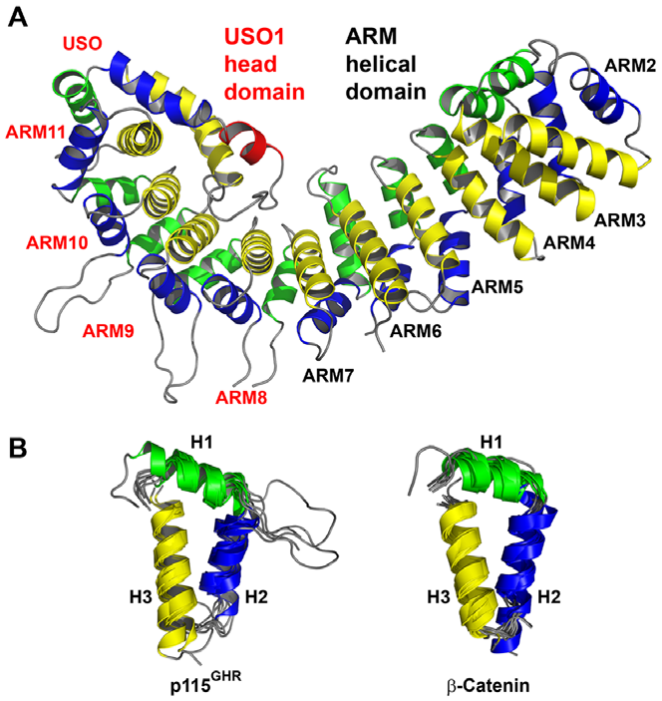
Crystal structure of human p115^GHR^ [13]. The color scheme of the ARM helices is the same as that in Figure 1. (**A**) The protein is composed of 11 ARM repeats and the USO element (repeat numbers are shown next to the repeats, in red for the USO1 head domain, in black for the armadillo helical domain). ARM1 is not visible in the structure of human p115^GHR^ but is partially resolved in the bovine p115^GHR^ structure. (**B**) A superimposition of the ARM repeats of human p115^GHR^ (excluding ARM2 due to a disordered H1) is shown on the left. For comparison, the ARM repeats of murine β-catenin are superimposed on the right.

Murine β-catenin (138–664) was the first structure of an ARM-repeat protein to have its structure solved [11], revealing that each ARM motif folds into a conserved three-dimensional structure consisting of three helices (H1, H2 and H3) that form a compact helical bundle with distinct features (Figures 1, 2). While H1 is the shortest helix containing approximately two turns, helices H2 and H3 comprise about three and four turns, respectively. Helices H2 and H3 share extensive hydrophobic interactions and are oriented in an antiparallel fashion, whereas H1 lies almost perpendicular to the remaining helices. Importantly, all H3 helices within the ARM fold decorate the superhelical groove of the solenoid structure, whereas helices H1 and H2 are located at the cylindrical outer surface [11].

Canonical ARM repeats possess a sequence of about 42 amino acids. Generally, the sequence similarity between the sequences of repeating ARM motifs within a single protein may be very low, but their similarity at the three-dimensional structure level tends to be high.

The ARM-repeat helix H1 contains five highly conserved residues within the universal consensus sequence [8]. Additionally, the Gly residue C-terminal of the ARM-repeat helix H1 is strongly conserved and mediates a distinct kink between H1 and H2 [11], [12]. ARM-repeat helix H2 possesses three highly conserved hydrophobic residues (usually Leu), one at the N-terminus of H2 and two consecutive hydrophobic residues in a block of eight conserved residues. ARM-repeat H3 contains ten conserved residues including a strongly conserved solvent exposed polar residue, most frequently an Asn at the C-terminus of the helix.

Recently, structural insight into vesicle tethering mediated by the ARM-repeat protein p115 has been provided [13], [14]. Although the two independently determined crystal structures are virtually identical, the two publications came to different conclusions regarding the classification of structural repeats present in p115. Whereas Striegl *et al.* [13] characterized p115 as an ARM-repeat protein, An *et al.* [14] suggested the presence of novel “tether repeats” (TR) in p115 and proposed that these tether repeats would also occur in a broad spectrum of other tether proteins.

In order to clarify this discrepancy, we here present a proper classification of the p115 ARM-motifs by combining both structural and sequence information. Additionally, in our analysis we observe no significant evidence that the p115 ARM-motif pattern is present in other tethering factors such as golgins GM130 and giantin.

## Analysis

### The Globular Head Region of p115: An ARM-Like Helical Conserved Structure

The human general vesicular transport factor p115 is a protein of the golgin family that gives identity and structure to the Golgi apparatus and is part of a complex protein network at the Golgi membrane [15]–[18]. p115 facilitates the tethering of transport vesicles inbound from the endoplasmic reticulum to the *cis*-Golgi membrane. The myosin-shaped protein forms stable homodimers and comprises a long central coiled-coil region (p115^CC^), a large N-terminal globular head domain (p115^GHR^) and a C-terminal acidic region [19], [20]. p115 is recruited to membranes by the guanosine triphosphatase (GTPase) Rab1a in a nucleotide-dependent manner and is among the best characterized representatives of long coiled-coil tethering factors [21]–[27].

Recently, the crystal structures of the human (Figure 2) and bovine p115^GHR^ were determined [13], [14]. Since human and bovine p115^GHR^ are more than 99% identical in their amino acid sequence, it comes as no surprise that the structure of human p115 (Protein Data Bank accession code 2W3C) is very similar to that of the bovine p115 (Protein Data Bank accession code 3GRL), yielding a Z-score of 47.6 for an alignment of 549 residues with a root-mean-square deviation of 1.1 Å by the DaliLite program [28]. The high structural similarity, confirmed by superposition of the α-carbon traces of the human and bovine p115, suggests that both proteins should share an identical structural classification.

**Figure 3:**
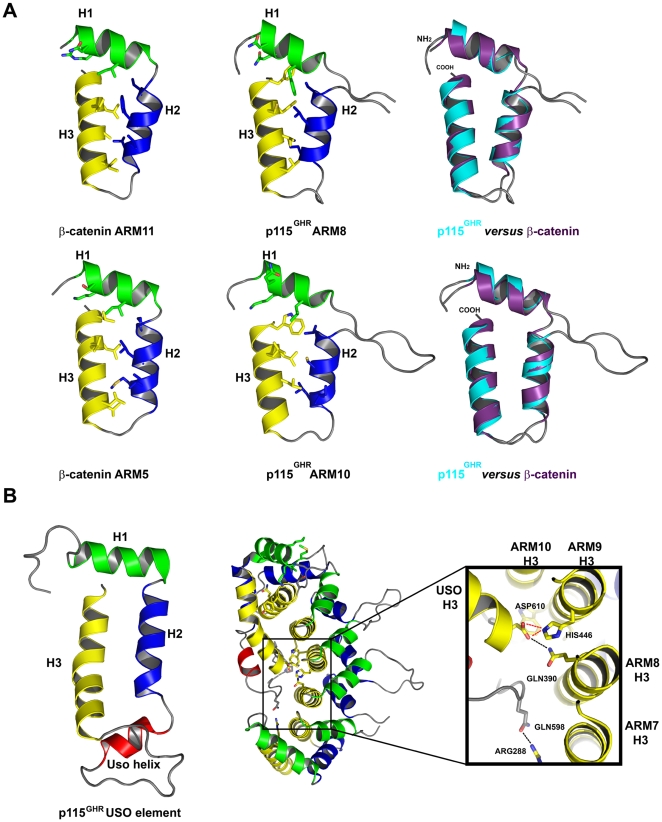
Comparison of repeat motif structures. (**A**) A comparison of p115^GHR^ ARM8/10 with β-catenin ARM11/5. The backbones are superimposed on the right. The individual repeats are shown on the left, with the side chains of the conserved consensus residues shown as sticks. (**B**) The C-terminal non-canonical USO element. Key residues that mediate interactions of the USO element with the superhelical groove are shown in stick representation on the right.

However, there are significant differences concerning the p115^GHR^ ARM-fold nomenclature and classification adopted in these publications. An *et al.* [14] claim that the p115^GHR^ solenoid is made up by a functionally specific TR motif. Striegl *et al.* [13], however, advance the view that this TR motif is actually a frame-shifted classical ARM repeat in which helix H1 of TR corresponds to H2 of ARM, H2 (TR) to H3 (ARM) and H3 (TR) to H1 (ARM). Accordingly, we argue that, on a sequence and structural level, p115^GHR^, indeed, belongs to the ARM-protein superfamily [13] (Figures 1, 2, 3).

In fact, the crystal structures of both the human and the bovine p115^GHR^ show that the protein consists of a multi-helical β-catenin-like ARM fold arranged in a regular right-handed superhelix. The published human p115^GHR^ structure included residues Asp54 to Tyr629 of p115 resulting in the assignment of the N-terminal armadillo repeat observed in the structure as ARM1 [13]. The globular head domain of bovine p115 [14] completes the full-length ARM fold of p115^GHR^ by an additional but incomplete (due to a disordered helix H1) ARM repeat at the N-terminus of the molecule. To facilitate a structural comparison, the ARM repeats in human p115^GHR^ have been renumbered such that ARM1 of Striegl *et al.* [13] is now labeled ARM2, and the last repeat preceding the ARM-like USO element is ARM11 (Figure 2a).

The N-terminal armadillo helical domain comprising ARM1 to ARM7 of p115^GHR^ (residues 1–342) is remarkably similar to members of different ARM-protein subfamilies. For example, an iterative sequence search of the database with this fragment using PSIBLAST [32] retrieves proteins containing ARM repeats at significant E-values (<0.005) already in the 2^nd^ iteration. On the contrary, the five C-terminal repeats of p115^GHR^ (residues 343–629, starting from ARM8) are not easily discernable as ARM-repeats at the sequence level. In fact, sequence analyses classify this region as a USO1 head domain (Figure 2), a domain that identifies a group of proteins described as general vesicular transport factors, transcytosis-associated proteins (TAP) or vesicle docking proteins [29]. A structure-based sequence alignment of p115^GHR^ and β-catenin ARM repeats, however, clearly shows that the conserved hydrophobic residues located in this region align very well, with the exception of the C-terminal four helices (USO element; Figure 1, Tables S1, S2). Thus, the ARM8-ARM11 repeats within the USO1 head domain are indeed armadillo repeats.

**Figure 4:**
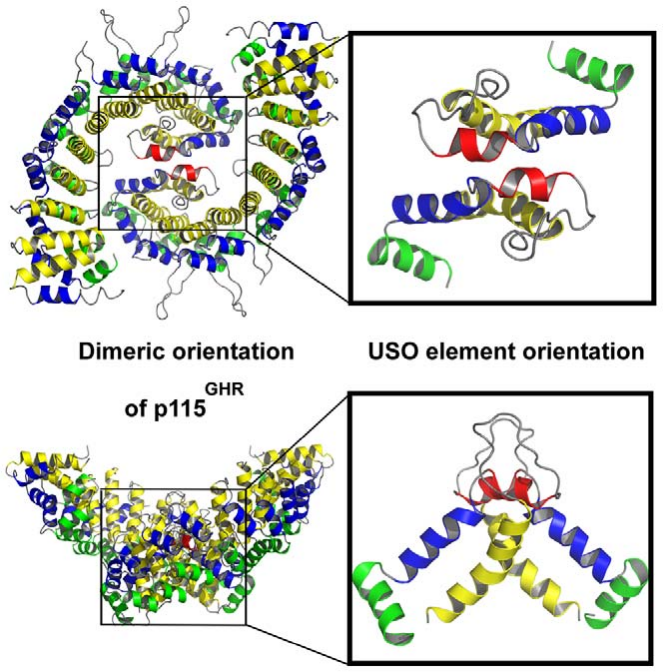
Dimeric organization of the USO element. The dimeric orientation of human p115^GHR^ is shown on the left, the orientation of the USO element on the right side accordingly.

The USO element folds back into the superhelical groove covering helices H3 of repeats ARM8-ARM11 [13] (Figures 2, 3b, 4). This possibly explains the described differences in sequence and structure between the N-terminal ARM domain and the C-terminal USO1 head domain of p115^GHR^. The interaction with the superhelical groove is mediated by hydrophobic interactions and a single salt bridge (Figure 3b, Table S3). In addition, the USO1 head domain displays large inter- and intra-repeat insertions (Figure 1, 2a). The ARM10 helix H1, for example, is connected to helix H2 by 15 residues, whereas the kink of these helices of ARM5 within β-catenin is mediated by a single glycine (Figure 1).

Despite these structural differences of the USO1 head domain, the superimposition of all p115^GHR^ repeats on the one hand and the superimposition of repeats of p115^GHR^ and β-catenin on the other hand reveals significant structural similarity and a common overall fold (Figure 2b, 3a). Thus, the repeats within the USO1 head domain are indeed ARM repeats with exception of the C-terminal USO element.

In summary, p115^GHR^ contains 11 ARM repeats. The last four C-terminal ARM repeats of p115GHR and the USO element form the USO1 head domain that reveals some sequence and structural alterations compared to the N-terminal classical ARM domain. These differences go along with the function of p115 in vesicular transport and tethering.

### The ARM Motifs of p115: Unique and Not Present in GM130 and Giantin

**Figure 5:**
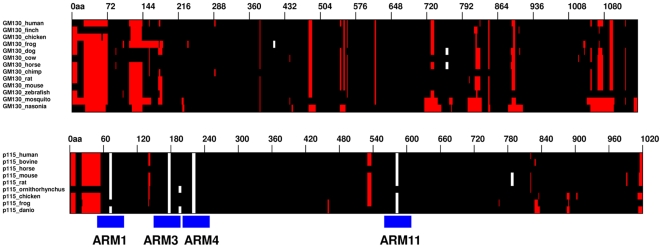
Search for alpha-rod repeats in p115, GM130 and homologs. Human sequences and representative orthologs were aligned, and the multiple sequence alignment (MSA) and hits to alpha-rods (from ARD) [33] were represented using BiasViz [33]. Basically, gaps are represented in red and aligned sequence in black unless a significant match to an alpha-rod was recorded (white). Top: MSA of human GM130 and orthologs from 12 species. Only three scattered matches are observed, and most sequences (including the human) did not have any significant match. Bottom: MSA of human p115 and orthologs from 7 species. Significant matches were observed in all but one ortholog for four ARM repeats (the human sequence showing the four of them).

Analysis of the globular head domain of bovine p115 by An *et al.* [14] led to the assumption that the p115^GHR^ repeats lack sequence conservation except for leucine-rich motifs, and, due to these characteristics, variable leucine-rich motifs for the helices H1, H2 and H3 were suggested [14]. Upon visual inspection, a pattern of leucine-rich residues separated by sequences of variable length, as found for p115^GHR^, was detected in other tether proteins that are involved in exocytic and endocytic trafficking [14], including the *cis*-Golgi golgins GM130 and giantin [reviewed in 16]. This sequence similarity was used for the characterization and classification of the TR motifs. However, iterative sequence searches with these proteins using PSIBLAST [28] did not support their similarity to p115 or to any protein with ARM-repeats. In order to make a more exhaustive analysis we collected orthologs of the GM130, giantin and p115 human proteins, and scanned them with ARD, which uses a neural network to detect ARM and other repeats forming alpha-rods [30]. Whereas four correct matches could be identified in the N-terminal part of most of the p115 homologs used, no such signal was obtained in human GM130, giantin (not shown), or their orthologs tested (Figure 5).

Additionally, we scanned ten golgin-related sequences (Golgin245, Golgin84, Gmap210, BicaudalD1, Iporin, Mical1, Rabenosyn5, Rabaptin5, EEA1, Rim3, Noc2) for alpha-rod repeats using the ARD server. None of the sequences was identified as containing such repeats: seven sequences received no single hit, and three (Rabaptin5, EEA1, Rim3) received one single hit above 0.8, whereas at least three such hits are taken as evidence for repeats.

## Discussion

Proteins within the different ARM subfamilies display a conserved architecture and provide a scaffold for the assembly of protein complexes with various functions. Generally, the identification of ARM repeats by sequence comparisons is relatively simple, the C-terminal region of p115^GHR^, however, demonstrates the difficulty to classify the protein as an ARM-fold protein just by sequence comparisons. This may explain why a structural annotation of bovine p115^GHR^ [14] invoked a new type of repeat (TR) which we find, however, neither required nor helpful in classifying this protein structure.

Crystal structure analysis revealed a special ARM-fold architecture of the p115^GHR^ C-terminal domain identified as the USO1 head domain, bearing large insertions and a unique USO element. This domain is inimitable among ARM-repeat proteins and defines proteins as vesicular transport factors. The unexpected ARM fold of the USO1 head domain of p115^GHR^ differs from the classical ARM fold, but structure-based sequence alignments advance a better understanding of how to unambiguously classify p115 as an ARM-protein superfamily member.

In conclusion, we propose to define a fourth subfamily of ARM-like proteins. Thus, besides the classical catenins, the p120^ctn^-related catenins and the proteins involved in nuclear import the new ARM subfamily is termed USO1 head domain-like and describes a group of proteins that are involved in vesicular transport and are conserved from yeast to human. Therefore, the globular head region of p115 is the first crystal structure of a member of the USO1 head domain-like ARM subfamily.

## Supporting Information

Table S1 Structure-based alignment of p115 and beta-catenin
(0.08 MB XLS)

Table S2 Structure-based alignment of p115 repeats
(0.05 MB XLS)

Table S3 Interactions of USO element with armadillo repeats of p115
(0.17 MB DOC)
