# Supplementary material for: Armadillo Motifs Involved in Vesicular Transport
Source: PLoS One. 2010 Feb 1;5(2):e8991. doi: 10.1371/journal.pone.0008991 (PMC2813876; doi:10.1371/journal.pone.0008991)
Supplement: Table S3 — Interactions of USO element with armadillo repeats of p115 (0.17 MB DOC) [file pone.0008991.s003.doc]

**Table S3**

**Hydrogen bonds**

**USO repeat (U) Distance[Å] p115^GHR^ (A)**

1   U:LYS 561[ N  ]   2.95   A:ASN 554[ OD1]

2   U:LYS 561[ NZ ]   3.25   A:TYR 550[ O  ]

3   U:LYS 561[ NZ ]   3.19   A:ASN 552[ O  ]

4   U:LYS 563[ N  ]   3.39   A:MET 560[ O  ]

5   U:LEU 564[ N  ]   2.97   A:MET 560[ O  ]

6   U:LYS 580[ NZ ]   3.60   A:GLN 538[ OE1]

7   U:GLN 598[ OE1]   2.62   A:ARG 288[ NH2]

8   U:PRO 604[ O  ]   3.06   A:GLN 489[ NE2]

9   U:MET 607[ O  ]   2.78   A:GLN 489[ NE2]

10   U:MET 607[ SD ]   3.88   A:CYS 439[ SG ]

11   U:ILE 608[ O  ]   2.81   A:TRP 438[ NE1]

12   U:ASP 610[ OD1]   3.15   A:GLN 390[ NE2]

13   U:ASP 610[ OD2]   3.03   A:HIS 446[ NE2]

**Salt bridges**

 1   U:ASP 610[ OD1]   3.35   A:HIS 446[ NE2]

2   U:ASP 610[ OD2]   3.03   A:HIS 446[ NE2]

**ASA**  Accessible Surface Area, Å²

**BSA**  Buried Surface Area, Å²

**Δ^i^G**  Solvation energy effect, kcal/mol

||||   Buried area percentage, one bar per 10%

**HSDC**Residues making **H**ydrogen/**D**isulphide bond, **S**alt bridge or **C**ovalent link

**USO repeat (U) HSDC ASA BSA** | | | |  **Δ^i^G**

1  U:LYS 561     H     198.56   132.80 ||||||||    -0.70

2  U:GLU 562            96.99     7.01 ||    0.05

3  U:LYS 563     H     149.03    61.11 |||||    0.21

4  U:LEU 564     H     123.36   123.08 |||||||||||  1.94

5  U:LYS 565            33.79     9.89 ||||    0.16

6  U:GLN 566           107.03     0.00    0.00

7  U:LEU 567            78.60    39.27 ||||||    0.63

8  U:ILE 568            26.17    26.01 |||||||||||  0.42

9  U:GLU 569            79.20     0.00    0.00

10  U:LYS 570           151.61     0.00    0.00

11  U:ARG 571           172.64    35.88 |||    0.24

12  U:ILE 572            85.31    64.84 |||||||||    1.04

13  U:GLY 573            16.65     0.00    -0.00

14  U:LYS 574            33.49     0.00    -0.00

15  U:GLU 575           120.29     0.00    0.00

16  U:ASN 576            65.90     4.67 ||     -0.08

17  U:PHE 577            51.17    51.17 |||||||||||  0.81

18  U:ILE 578            40.01     0.00    -0.00

19  U:GLU 579           100.33     0.00     -0.00

20  U:LYS 580     H     103.85    85.24 |||||||||   -0.42

21  U:LEU 581            36.01    34.84 |||||||||||  0.52

22  U:GLY 582            7.87     0.00    0.00

23  U:PHE 583           108.09    77.65 ||||||||    1.17

24  U:ILE 584            53.03    53.03 |||||||||||  0.85

25  U:SER 585            37.80     0.00    0.00

26  U:LYS 586           155.20     0.00    -0.00

27  U:HIS 587            61.87    21.97 |||||    0.16

28  U:GLU 588           142.69     0.00    0.00

29  U:LEU 589            42.00     0.00    0.00

30  U:TYR 590            25.15     0.00    -0.00

31  U:SER 591            76.06     0.00    -0.00

32  U:ARG 592           137.38     0.00    0.00

33  U:ALA 593            17.99     0.00    -0.00

34  U:SER 594            48.41     0.00    -0.00

35  U:GLN 595           120.92     0.00    0.00

36  U:LYS 596           138.41    16.33 ||    -0.28

37  U:PRO 597           106.35    80.82 |||||||||    1.02

38  U:GLN 598     H     140.52    76.72 ||||||    -0.20

39  U:PRO 599           101.24    81.45 |||||||||  1.16

40  U:ASN 600            81.86     0.00    0.00

41  U:PHE 601            68.87    37.07 ||||||    0.34

42  U:PRO 602            98.74     1.88 |    0.00

43  U:SER 603            45.94     7.35 |||    0.12

44  U:PRO 604     H     106.20    61.09 |||||||    0.70

45  U:GLU 605           143.83     1.69 |    0.02

46  U:TYR 606            92.93     0.00    0.00

47  U:MET 607     H     130.34   128.17 |||||||||||  2.54

48  U:ILE 608     H     105.05    88.75 |||||||||    0.63

49  U:PHE 609            23.86    17.97 |||||||||    0.28

50  U:ASP 610     HS     91.15    77.97 ||||||||||  -0.08

51  U:HIS 611            23.57     0.58 |    -0.02

52  U:GLU 612           105.69    76.19 ||||||||    0.55

53  U:PHE 613            88.33    80.17 ||||||||||  1.28

54  U:THR 614            2.16     0.00    -0.00

55  U:LYS 615            98.06     8.54 ||    -0.32

56  U:LEU 616            84.44    63.37 |||||||||  1.01

57  U:VAL 617             8.87     8.54 ||||||||||| 0.14

58  U:LYS 618           168.54     0.00    -0.00

59  U:GLU 619           117.59    16.14 ||    -0.19

60  U:LEU 620            62.31    56.95 ||||||||||  0.91

61  U:GLU 621            44.08     2.59 ||    0.03

62  U:GLY 622            51.29     0.00    0.00

63  U:VAL 623            89.44    32.26 |||||    0.49

64  U:ILE 624            63.84    63.05 |||||||||||  1.01

65  U:THR 625            43.37     0.00    -0.00

66  U:LYS 626           158.51     0.00    -0.00

67  U:ALA 627            57.84    28.59 ||||||    0.45

68  U:ILE 628            18.48    17.65 |||||||||||  0.28

69  U:TYR 629           157.74     0.00    -0.00

**p115^GHR^ (A) HSDC ASA BSA** | | | | **Δ^i^G**

1  A:LEU  55           139.52     0.00    0.00

2  A:GLU  56            95.25     0.00     0.00

3  A:VAL  57           100.07     0.00     -0.00

4  A:GLY  58            29.74     0.00     -0.00

5  A:ILE  59            27.64     0.00     -0.00

6  A:GLN  60            50.03     0.00    0.00

7  A:ALA  61            64.86     0.00     -0.00

8  A:MET  62             2.34     0.00     -0.00

9  A:GLU  63           125.19     0.00     -0.00

10  A:HIS  64           106.28     0.00    0.00

11  A:LEU  65             9.88     0.00    0.00

12  A:ILE  66            18.14     0.00    -0.00

13  A:HIS  67           100.48     0.00    -0.00

14  A:VAL  68            18.08     0.00    -0.00

15  A:LEU  69            4.35     0.00    -0.00

16  A:GLN  70            92.02     0.00    -0.00

17  A:THR  71            88.62     0.00    -0.00

18  A:ASP  72            61.99     0.00    -0.00

19  A:ARG  73            88.45     0.00    -0.00

20  A:SER  74           124.40     0.00    0.00

21  A:ASP  75            63.00     0.00    -0.00

22  A:SER  76            29.64     0.00    -0.00

23  A:GLU  77           123.59     0.00    -0.00

24  A:ILE  78            56.21     0.00    0.00

25  A:ILE  79            2.17     0.00    0.00

26  A:GLY  80             8.11     0.00    0.00

27  A:TYR  81           124.96     0.00    -0.00

28  A:ALA  82             0.17     0.00    -0.00

29  A:LEU  83             0.00     0.00    -0.00

30  A:ASP  84            60.51     0.00    0.00

31  A:THR  85            34.00     0.00    -0.00

32  A:LEU  86            0.00     0.00    -0.00

33  A:TYR  87            59.84     0.00    -0.00

34  A:ASN  88            96.86     0.00    -0.00

35  A:ILE  89            44.20     0.00    -0.00

36  A:ILE  90            0.17     0.00    0.00

37  A:SER  91            46.62     0.00    -0.00

38  A:ASN  92           153.15     0.00     -0.00

39  A:SER 111            61.74     0.00    0.00

40  A:GLN 112           125.36     0.00    -0.00

41  A:PHE 113            47.82     0.00    -0.00

42  A:THR 114            0.00     0.00    -0.00

43  A:GLU 115            83.18     0.00    0.00

44  A:ILE 116            87.04     0.00    -0.00

45  A:PHE 117             3.80     0.00    -0.00

46  A:ILE 118            11.70     0.00    0.00

47  A:LYS 119           168.33     0.00    0.00

48  A:GLN 120           109.61     0.00    -0.00

49  A:GLN 121           103.23     0.00    -0.00

50  A:GLU 122           102.09     0.00    -0.00

51  A:ASN 123            14.33     0.00    -0.00

52  A:VAL 124             0.00     0.00    0.00

53  A:THR 125            55.42     0.00    -0.00

54  A:LEU 126            27.80     0.00    0.00

55  A:LEU 127             1.13     0.00    0.00

56  A:LEU 128             2.36     0.00    0.00

57  A:SER 129            30.17     0.00    -0.00

58  A:LEU 130            1.51     0.00    0.00

59  A:LEU 131            8.96     0.00    -0.00

60  A:GLU 132            88.12     0.00    -0.00

61  A:GLU 133            64.78     0.00    0.00

62  A:PHE 134           155.05     0.00    -0.00

63  A:ASP 135            55.61     0.00    -0.00

64  A:PHE 136            69.53     0.00    -0.00

65  A:HIS 137            70.82     0.00    -0.00

66  A:VAL 138             2.63     0.00    0.00

67  A:ARG 139             8.29     0.00    -0.00

68  A:TRP 140            80.53     0.00    0.00

69  A:PRO 141             6.69     0.00    0.00

70  A:GLY 142            0.00     0.00    0.00

71  A:VAL 143            0.17     0.00    -0.00

72  A:LYS 144            36.57     0.00    0.00

73  A:LEU 145            0.00     0.00    -0.00

74  A:LEU 146            0.00     0.00    -0.00

75  A:THR 147            14.69     0.00    -0.00

76  A:SER 148            12.01     0.00    -0.00

77  A:LEU 149             0.00     0.00    -0.00

78  A:LEU 150             3.31     0.00    -0.00

79  A:LYS 151           153.82     0.00    -0.00

80  A:GLN 152            60.98     0.00    -0.00

81  A:LEU 153            27.58     0.00    -0.00

82  A:GLY 154            10.31     0.00    -0.00

83  A:PRO 155           105.29     0.00    0.00

84  A:GLN 156            89.51     0.00    -0.00

85  A:VAL 157             0.00     0.00    0.00

86  A:GLN 158            6.60     0.00    -0.00

87  A:GLN 159            86.17     0.00    -0.00

88  A:ILE 160            14.49     0.00    -0.00

89  A:ILE 161            0.24     0.00    0.00

90  A:LEU 162            63.14     0.00    -0.00

91  A:VAL 163           112.01     0.00    0.00

92  A:SER 164            11.74     0.00    -0.00

93  A:PRO 165           110.98     0.00    0.00

94  A:MET 166           143.47     0.00    -0.00

95  A:GLY 167             2.49     0.00    -0.00

96  A:VAL 168             6.97     0.00    -0.00

97  A:SER 169            30.44     0.00    0.00

98  A:ARG 170            58.32     0.00    0.00

99  A:LEU 171             2.34     0.00    -0.00

100  A:MET 172            2.67     0.00    -0.00

101  A:ASP 173            79.05     0.00    0.00

102  A:LEU 174            1.34     0.00    -0.00

103  A:LEU 175            8.66     0.00    -0.00

104  A:ALA 176            65.20     0.00    0.00

105  A:ASP 177            17.30     0.00    0.00

106  A:SER 178           110.06     0.00    0.00

107  A:ARG 179            80.51     0.00    -0.00

108  A:GLU 180            94.71     0.00    0.00

109  A:VAL 181            74.48     0.00    0.00

110  A:ILE 182            0.00     0.00    0.00

111  A:ARG 183            24.96     0.00    -0.00

112  A:ASN 184            35.88     0.00    -0.00

113  A:ASP 185            33.61     0.00    -0.00

114  A:GLY 186             0.00     0.00    0.00

115  A:VAL 187             0.84     0.00    -0.00

116  A:LEU 188            68.60     0.00    -0.00

117  A:LEU 189             0.00     0.00    -0.00

118  A:LEU 190             0.00     0.00    -0.00

119  A:GLN 191            25.82     0.00    -0.00

120  A:ALA 192            12.19     0.00    -0.00

121  A:LEU 193            0.00     0.00    -0.00

122  A:THR 194            0.00     0.00    -0.00

123  A:ARG 195           172.32     0.00    -0.00

124  A:SER 196           107.87     0.00    -0.00

125  A:ASN 197            23.67     0.00    0.00

126  A:GLY 198            35.25     0.00    0.00

127  A:ALA 199            51.55     0.00    -0.00

128  A:ILE 200             0.00     0.00    0.00

129  A:GLN 201            13.27     0.00    -0.00

130  A:LYS 202            69.55     0.00    -0.00

131  A:ILE 203            47.87     0.00    0.00

132  A:VAL 204             0.17     0.00    0.00

133  A:ALA 205             1.42     0.00    0.00

134  A:PHE 206           136.88     0.00    0.00

135  A:GLU 207            61.16     0.00    0.00

136  A:ASN 208            66.85     0.00    -0.00

137  A:ALA 209            0.00     0.00    0.00

138  A:PHE 210            1.00     0.00    -0.00

139  A:GLU 211            72.04     0.00    -0.00

140  A:ARG 212            60.66     0.00    -0.00

141  A:LEU 213            0.00     0.00    -0.00

142  A:LEU 214            2.94     0.00    0.00

143  A:ASP 215            54.74     0.00    -0.00

144  A:ILE 216            10.71     0.00    0.00

145  A:ILE 217             0.00     0.00    0.00

146  A:SER 218            60.46     0.00    0.00

147  A:GLU 219           124.67     0.00    -0.00

148  A:GLU 220            53.24     0.00    -0.00

149  A:GLY 221            36.93     0.00    0.00

150  A:ASN 222            31.80     0.00    -0.00

151  A:SER 223             5.93     0.00    -0.00

152  A:ASP 224           100.31     0.00    -0.00

153  A:GLY 225             9.83     0.00     -0.00

154  A:GLY 226            30.93     0.00    0.00

155  A:ILE 227           116.91     0.00    0.00

156  A:VAL 228            24.43     0.00    -0.00

157  A:VAL 229             0.51     0.00    0.00

158  A:GLU 230            35.65     0.00    0.00

159  A:ASP 231            17.10     0.00     -0.00

160  A:CYS 232            0.00     0.00    0.00

161  A:LEU 233            0.12     0.00     -0.00

162  A:ILE 234            50.24     0.00    -0.00

163  A:LEU 235             0.17     0.00    0.00

164  A:LEU 236             0.84     0.00    -0.00

165  A:GLN 237            43.82     0.00    0.00

166  A:ASN 238            28.57     0.00    0.00

167  A:LEU 239             0.00     0.00    -0.00

168  A:LEU 240            0.50     0.00    0.00

169  A:LYS 241           118.53     0.00    0.00

170  A:ASN 242           142.25     0.00    -0.00

171  A:ASN 243            6.94     0.00    -0.00

172  A:ASN 244            82.12     0.00    -0.00

173  A:SER 245            61.75     0.00    0.00

174  A:ASN 246            0.61     0.00    0.00

175  A:GLN 247            9.23     0.00    -0.00

176  A:ASN 248            77.61     0.00    -0.00

177  A:PHE 249            74.83     0.00    -0.00

178  A:PHE 250            0.94     0.00    -0.00

179  A:LYS 251            49.30     0.00    0.00

180  A:GLU 252           117.67     0.00    0.00

181  A:GLY 253            35.72     0.00     -0.00

182  A:SER 254            64.04     0.00    0.00

183  A:TYR 255            25.72     0.00    -0.00

184  A:ILE 256            5.02     0.00    0.00

185  A:GLN 257           100.83     0.00    0.00

186  A:ARG 258            77.61     0.00    -0.00

187  A:MET 259            1.00     0.00    -0.00

188  A:LYS 260            60.16     0.00    -0.00

189  A:PRO 261            64.91     0.00    0.00

190  A:TRP 262            7.35     0.00    -0.00

191  A:PHE 263            0.62     0.00    -0.00

192  A:GLU 264            96.15     0.00    -0.00

193  A:VAL 265            84.70     0.00    -0.00

194  A:GLY 271           126.35     0.00    -0.00

195  A:TRP 272            53.11     0.00    0.00

196  A:SER 273            67.63     0.00    -0.00

197  A:ALA 274            88.24     0.00    -0.00

198  A:GLN 275            52.98     0.00    0.00

199  A:LYS 276            26.02     0.00    -0.00

200  A:VAL 277            29.21     0.00    -0.00

201  A:THR 278            48.57     0.00    0.00

202  A:ASN 279            0.00     0.00    0.00

203  A:LEU 280             6.07     0.00    -0.00

204  A:HIS 281            41.75     0.00    0.00

205  A:LEU 282            26.36     0.00    0.00

206  A:MET 283             0.00     0.00    -0.00

207  A:LEU 284             2.69     0.00    -0.00

208  A:GLN 285            88.00     0.00    0.00

209  A:LEU 286            0.67     0.00     -0.00

210  A:VAL 287            0.00     0.00    0.00

211  A:ARG 288     H      67.80    23.08 ||||    -0.44

212  A:VAL 289            20.89     0.00    -0.00

213  A:LEU 290            0.37     0.00    -0.00

214  A:VAL 291            1.10     0.00    0.00

215  A:SER 292            22.06     0.00    0.00

216  A:PRO 293            71.87    21.27 ||||    0.34

217  A:THR 294           123.42    17.79 ||    -0.17

218  A:ASN 295            15.00     0.00    0.00

219  A:PRO 296            66.57     0.00    0.00

220  A:PRO 297           117.03     0.00    0.00

221  A:GLY 298            47.60     0.00    -0.00

222  A:ALA 299            30.63     0.00    0.00

223  A:THR 300            15.98     0.00     -0.00

224  A:SER 301            46.11     0.00    0.00

225  A:SER 302            33.53     0.00    0.00

226  A:CYS 303             3.33     0.00    -0.00

227  A:GLN 304            4.98     0.00    -0.00

228  A:LYS 305           114.58     0.00    -0.00

229  A:ALA 306            28.49     0.00    0.00

230  A:MET 307            0.00     0.00     -0.00

231  A:PHE 308            48.58     0.00    -0.00

 232  A:GLN 309          136.32     0.00    -0.00

233  A:CYS 310            35.84     0.00    -0.00

234  A:GLY 311            18.05     0.00    0.00

235  A:LEU 312            0.00     0.00    -0.00

236  A:LEU 313             0.00     0.00     -0.00

237  A:GLN 314            49.18     0.00    0.00

238  A:GLN 315            30.35     0.00    -0.00

239  A:LEU 316             0.00     0.00    -0.00

240  A:CYS 317             2.25     0.00    -0.00

241  A:THR 318            60.91     0.00    0.00

242  A:ILE 319            2.34     0.00    -0.00

243  A:LEU 320            42.20     0.00    0.00

244  A:MET 321            88.11     0.00    0.00

245  A:ALA 322            30.70     0.00    -0.00

246  A:THR 323           134.10     0.00    -0.00

247  A:GLY 324            68.00     0.00    0.00

248  A:VAL 325            17.18     0.00    0.00

249  A:PRO 326            37.79     0.00    0.00

250  A:ALA 327            74.08     0.00    -0.00

251  A:ASP 328            69.33     0.00    -0.00

252  A:ILE 329             0.00     0.00    0.00

253  A:LEU 330            49.72     0.00    0.00

254  A:THR 331            5.62     0.00    -0.00

255  A:GLU 332            47.41     0.61 |    -0.01

256  A:THR 333            0.12     0.00    -0.00

257  A:ILE 334             9.12     0.00    0.00

258  A:ASN 335            22.78    14.80 |||||||    -0.12

259  A:THR 336             0.00     0.00    -0.00

260  A:VAL 337             0.00     0.00    0.00

261  A:SER 338            0.00     0.00    -0.00

262  A:GLU 339            11.42     1.22 ||    -0.02

263  A:VAL 340            0.00     0.00    0.00

264  A:ILE 341            0.00     0.00    0.00

265  A:ARG 342            78.93    34.04 |||||    -0.65

266  A:GLY 343            39.79     0.00    -0.00

267  A:CYS 344            11.31     0.00    0.00

268  A:GLN 345            72.56     0.00    0.00

269  A:VAL 346            76.45     0.00    -0.00

270  A:ASN 347             0.25     0.00    0.00

271  A:GLN 348            1.84     0.00    -0.00

272  A:ASP 349            55.52     0.00    -0.00

273  A:TYR 350            61.21     0.00    -0.00

274  A:PHE 351            3.28     0.00    -0.00

275  A:ALA 352            30.23     0.00    -0.00

276  A:SER 353            72.62     0.00    -0.00

277  A:VAL 354            19.09     0.00    -0.00

278  A:ASN 355            83.85     0.00    -0.00

279  A:ALA 356            25.62     0.00    0.00

280  A:PRO 357           116.15     0.00    -0.00

281  A:SER 358            84.13     0.00    -0.00

282  A:PRO 360            67.80     0.00    0.00

283  A:PRO 361            89.48     0.00    -0.00

284  A:ARG 362            70.01     0.00    -0.00

285  A:PRO 363            54.76     0.00    -0.00

286  A:ALA 364             6.53     0.00    -0.00

287  A:ILE 365            13.71     0.00    0.00

288  A:VAL 366            9.12     0.00    0.00

289  A:VAL 367            59.88     0.00    0.00

290  A:LEU 368            7.85     0.00    0.00

291  A:LEU 369             0.17     0.00    0.00

292  A:MET 370            54.70     0.00    0.00

293  A:SER 371            15.13     0.00    -0.00

294  A:MET 372             0.17     0.00    0.00

295  A:VAL 373            31.25     0.00    0.00

296  A:ASN 374            64.60     0.00    -0.00

297  A:GLU 375            91.01     0.00    0.00

298  A:ARG 376           204.47     0.00    0.00

299  A:GLN 377            60.47     0.00    -0.00

300  A:PRO 378            72.23     0.00    0.00

301  A:PHE 379           135.45    98.90 ||||||||    1.58

302  A:VAL 380            90.65     0.00    -0.00

303  A:LEU 381            11.97     0.00    -0.00

304  A:ARG 382            8.99     0.00    0.00

305  A:CYS 383            56.82    42.55 ||||||||    1.21

306  A:ALA 384            2.00     0.00    0.00

307  A:VAL 385             4.97     0.00    0.00

308  A:LEU 386            14.55    13.38 ||||||||||   0.21

309  A:TYR 387            57.57    55.13 |||||||||||  0.70

310  A:CYS 388             0.00     0.00    0.00

311  A:PHE 389             0.00     0.00    -0.00

312  A:GLN 390     H      36.74    24.77 ||||||||    -0.34

313  A:CYS 391            1.48     0.00    0.00

314  A:PHE 392            4.38     0.00    -0.00

315  A:LEU 393            2.80     0.00    -0.00

316  A:TYR 394            72.23    22.52 ||||    0.17

317  A:LYS 395           138.03     0.00    -0.00

318  A:ASN 396             1.73     0.00    -0.00

319  A:GLN 397           107.80     0.00    -0.00

320  A:LYS 398           112.14     0.00    0.00

321  A:GLY 399             0.33     0.00    -0.00

322  A:GLN 400            7.32     0.00    -0.00

323  A:GLY 401            13.09     0.00     0.00

324  A:GLU 402            80.81     0.00    0.00

325  A:ILE 403            6.31     0.00    0.00

326  A:VAL 404             0.00     0.00    0.00

327  A:SER 405            63.56     0.00    -0.00

328  A:THR 406            37.46     0.00    0.00

329  A:LEU 407            14.07     0.00    -0.00

330  A:LEU 408            37.76     0.00    0.00

331  A:PRO 409           115.81     0.00    -0.00

332  A:SER 410           110.41     0.00    0.00

333  A:THR 411           116.88     0.00    -0.00

334  A:ILE 412           163.99     0.00    0.00

335  A:ASP 413            71.88     0.00    -0.00

336  A:ALA 414           107.45     0.00    -0.00

337  A:THR 415            83.30     0.00    0.00

338  A:GLY 416            83.99     0.00    -0.00

339  A:ASN 417            92.70     0.00    0.00

340  A:SER 418            78.23     0.00    -0.00

341  A:VAL 419            51.90     0.00    -0.00

342  A:SER 420            42.22     0.00    0.00

343  A:ALA 421             5.79     0.00    -0.00

344  A:GLY 422             1.41     0.00    0.00

345  A:GLN 423           124.89     0.00    -0.00

346  A:LEU 424            28.62     0.00    0.00

347  A:LEU 425             0.00     0.00    -0.00

348  A:CYS 426            25.30     0.00    0.00

349  A:GLY 427            44.83     0.00    -0.00

350  A:GLY 428            0.00     0.00     0.00

351  A:LEU 429            4.04     0.00    -0.00

352  A:PHE 430            70.17     0.00    0.00

353  A:SER 431            34.00     0.00    0.00

354  A:THR 432           130.74     0.00    -0.00

355  A:ASP 433            25.90     0.00    -0.00

356  A:SER 434             9.91     0.00    -0.00

357  A:LEU 435            58.28    28.83 ||||||    0.46

358  A:SER 436             0.49     0.00    -0.00

359  A:ASN 437             7.28     0.00    -0.00

360  A:TRP 438     H      76.59    75.34 |||||||||||  0.99

361  A:CYS 439     H      12.54    10.39 |||||||||    0.42

362  A:ALA 440            0.00     0.00    0.00

363  A:ALA 441            0.00     0.00    0.00

364  A:VAL 442            12.73    10.89 ||||||||||   0.17

365  A:ALA 443             0.12     0.00    0.00

366  A:LEU 444             0.00     0.00    -0.00

367  A:ALA 445             6.53     0.00    -0.00

368  A:HIS 446     HS     44.55    32.39 ||||||||     0.36

369  A:ALA 447            0.00     0.00    0.00

370  A:LEU 448            0.00     0.00    -0.00

371  A:GLN 449            59.62    26.56 |||||    -0.22

372  A:GLU 450           135.89     1.47 |    -0.02

373  A:ASN 451            7.03     0.00    0.00

374  A:ALA 452            50.81     0.00     -0.00

375  A:THR 453            89.13     0.00     0.00

376  A:GLN 454            26.03     0.00    -0.00

377  A:LYS 455            8.53     0.00    -0.00

378  A:GLU 456            73.05     0.00    0.00

379  A:GLN 457            56.28     0.00    -0.00

380  A:LEU 458             0.37     0.00    -0.00

381  A:LEU 459            21.76     0.00    -0.00

382  A:ARG 460           148.67     0.00     -0.00

383  A:VAL 461             4.29     0.00    0.00

384  A:GLN 462           114.01     0.00     -0.00

385  A:LEU 463            29.74     0.00    0.00

386  A:ALA 464           103.00     0.00    -0.00

387  A:THR 465            68.04     0.00    -0.00

388  A:SER 466            63.31     0.00    -0.00

389  A:ILE 467           189.96     0.00    0.00

390  A:GLY 468            75.69     0.00    0.00

391  A:ASN 469            99.76     0.00    0.00

392  A:PRO 470            98.07     0.00    0.00

393  A:PRO 471            80.35     0.00    0.00

394  A:VAL 472            31.29     0.00    0.00

395  A:SER 473            29.74     0.00    -0.00

396  A:LEU 474             0.15     0.00    -0.00

397  A:LEU 475            8.03     0.00    -0.00

398  A:GLN 476            20.00     0.00    -0.00

399  A:GLN 477            41.03     0.00    0.00

400  A:CYS 478            0.00     0.00    0.00

401  A:THR 479             5.75     0.00    0.00

402  A:ASN 480            50.15     0.00    0.00

403  A:ILE 481            21.60     0.00    -0.00

404  A:LEU 482             2.16     0.00    -0.00

405  A:SER 483            77.67     0.00    0.00

406  A:GLN 484           100.22     0.00    0.00

407  A:GLY 485            89.11     0.00    -0.00

408  A:SER 486            23.12     0.00    0.00

409  A:LYS 487           126.24     0.00    0.00

410  A:ILE 488            70.96    17.07 |||    0.27

411  A:GLN 489     H      70.44    48.34 ||||||||    -0.39

412  A:THR 490             8.50     0.00    -0.00

413  A:ARG 491            17.98     0.00    0.00

414  A:VAL 492            17.42    16.25 ||||||||||  0.26

415  A:GLY 493            0.00     0.00    0.00

416  A:LEU 494            0.67     0.00    -0.00

417  A:LEU 495            0.00     0.00    -0.00

418  A:MET 496            63.26    60.25 |||||||||||  1.99

419  A:LEU 497             0.00     0.00    -0.00

420  A:LEU 498             0.00     0.00    -0.00

421  A:CYS 499            10.49    10.49 ||||||||||| 0.17

422  A:THR 500            30.85    22.63 ||||||||    0.05

423  A:TRP 501            0.00     0.00    0.00

424  A:LEU 502            0.00     0.00    -0.00

425  A:SER 503            28.74    23.42 |||||||||    0.01

426  A:ASN 504           105.28    14.80 ||    -0.17

427  A:CYS 505             0.83     0.00    -0.00

428  A:PRO 506            45.28     0.00    0.00

429  A:ILE 507            73.18     0.00    0.00

430  A:ALA 508             0.00     0.00    0.00

431  A:VAL 509             1.51     0.00    0.00

432  A:THR 510            31.29     0.00    -0.00

433  A:HIS 511            93.21     0.00    -0.00

434  A:PHE 512            0.33     0.00    -0.00

435  A:LEU 513            6.86     3.19 ||||||    0.05

436  A:HIS 514           135.42     0.00    0.00

437  A:ASN 515            52.28     0.00    0.00

438  A:SER 516            84.40     0.00    -0.00

439  A:ALA 517            65.83     0.00    0.00

440  A:ASN 518             2.38     0.00    0.00

441  A:VAL 519            22.23    21.73 |||||||||||  0.35

442  A:PRO 520            72.83     2.17 |    0.03

443  A:PHE 521            36.66     0.00    -0.00

444  A:LEU 522             0.00     0.00    -0.00

445  A:THR 523            69.08    60.92 ||||||||||  0.52

446  A:GLY 524            30.81     0.00    -0.00

447  A:GLN 525            35.78     0.00    -0.00

448  A:ILE 526            56.63    56.63 |||||||||||  0.49

449  A:ALA 527            83.71    42.40 ||||||    0.56

450  A:GLU 528           106.71     0.63 |    0.01

451  A:ASN 529           149.43     9.01 ||    0.05

452  A:LEU 530            37.08     0.00    0.00

453  A:GLY 531            56.13     0.00    0.00

454  A:GLU 532            69.25     0.00    -0.00

455  A:GLU 533           136.99     0.00    0.00

456  A:GLU 534            45.08     0.00    0.00

457  A:GLN 535           111.87    52.44 ||||||    -0.20

458  A:LEU 536            98.07    93.37 |||||||||||  1.49

459  A:VAL 537             2.18     0.00    0.00

460  A:GLN 538     H      25.91    23.74 ||||||||||  -0.02

461  A:GLY 539            41.67    41.67 |||||||||||  0.49

462  A:LEU 540            25.09    23.59 ||||||||||   0.38

463  A:CYS 541            0.33     0.00    0.00

464  A:ALA 542            44.11    44.11 ||||||||||| 0.57

465  A:LEU 543            98.79    98.79 |||||||||||  1.54

466  A:LEU 544            0.00     0.00    -0.00

467  A:LEU 545            40.73    39.22 |||||||||||  0.63

468  A:GLY 546            33.98    33.36 |||||||||||  0.43

469  A:ILE 547            17.49    17.49 |||||||||||  0.28

470  A:SER 548             2.17     2.17 |||||||||||  0.03

471  A:ILE 549           124.89   124.05 |||||||||||  1.74

472  A:TYR 550     H     173.11    95.67 |||||||    0.93

473  A:PHE 551            42.78    17.64 |||||    0.28

474  A:ASN 552     H      35.47    22.25 |||||||    -0.13

475  A:ASP 553            79.54     0.00    0.00

476  A:ASN 554     H      89.25    21.48 |||    -0.25

477  A:SER 555            83.34     0.00    0.00

478  A:LEU 556            28.58     0.00    0.00

479  A:GLU 557            94.50     3.74 |    -0.04

480  A:SER 558            86.64    18.79 |||  -0.21

481  A:TYR 559            74.01    46.07 |||||||    0.70

482  A:MET 560     H     150.94    74.95 ||||||    0.12
